# Examining the Relationship Between Perceived Value and Movie Consumption Behavioral Intention: The Mediating Role of Satisfaction

**DOI:** 10.3390/bs16040556

**Published:** 2026-04-08

**Authors:** Nicong Zhao, Xia Zhu, Xiaoquan Pan

**Affiliations:** 1 College of Art, Zhejiang Normal University, Jinhua 321004, China; 20245586@zjnu.edu.cn; 2 Academic Affairs Office, Shanghai Jian Qiao University, Shanghai 201306, China; 11038@gench.edu.cn; 3 Xingzhi College, Zhejiang Normal University, Jinhua 321004, China; 4 College of Foreign Languages, Shanghai Jian Qiao University, Shanghai 201306, China

**Keywords:** perceived value, satisfaction, movie consumption, consumption behavioral intention

## Abstract

This study addressed a critical gap in understanding the drivers of movie consumption during digital transformation and streaming platform proliferation. It examined the direct effects of three core dimensions—social value, functional value, and emotional value—on movie consumption behavioral intention, alongside the mediating mechanism of satisfaction. Data were collected via questionnaire surveys administered to cinema audiences in Eastern China and through *Wenjuanxing* online platform, yielding 1089 valid responses. Statistical analysis was conducted using SPSS 26.0, and Structural Equation Modeling (SEM) was performed employing AMOS 26.0. Findings indicate significant positive direct effects of social value and emotional value on movie consumption behavioral intention. Furthermore, these value dimensions indirectly enhance movie consumption behavioral intention through the mediating influence of satisfaction. In contrast, functional value demonstrates no significant direct effect on either movie consumption behavioral intention or satisfaction. Satisfaction serves as a significant mediator in the relationships between both social value and emotional value, and movie consumption behavioral intention. This study elaborated the distinct pathways through which varied perceived value dimensions operate and empirically validates the mediating role of satisfaction within movie consumption decision-making. For the movie industry, these insights suggest prioritizing social engagement and emotional resonance to optimize offerings, establishing dynamic satisfaction monitoring, and designing member incentives targeting these values to foster sustained behavioral activation. This provides empirically grounded guidance for refining marketing strategies and experiential enhancements.

## 1. Introduction

With the global digital transformation of the film industry and proliferation of streaming platforms, consumer choices regarding theatrical movie consumption have undergone profound shifts. Movie consumption is no longer solely assessed on the intrinsic quality of content; it has expanded into a multidimensional value perception process incorporating considerations like time cost, service experience, emotional resonance, and social attributes (Kang et al., 2022). In competitive markets, understanding the drivers of sustained consumer engagement is essential for producers, distributors, and streaming platforms to optimize strategies. This understanding provides essential guidance for optimizing product design, enhancing marketing effectiveness, and constructing core competencies. The overall perceived value formed by consumers based on the movie product and associated services is considered as a core antecedent variable influencing their ultimate behavioral intention within this process.

Although extant research in service marketing and consumer behavior widely acknowledges the significant positive impact of perceived value on behavioral intention (e.g., purchase intention, loyalty, word-of-mouth) (C. Wang et al., 2023), and identifies the vital mediating role of customer satisfaction (Hussain, 2016), critical questions remain underexplored within the specific context of movie consumption—a service characterized by its cultural product attributes and experiential–emotional nature. Current literature shows limitations regarding the unique components and measurement of perceived value within this industry context, such as emotional and social value dimensions (Hasson et al., 2008). While preliminary studies have established the influencing components in certain aspects of the movie consumption pathway (De Luca, 2017), there remains a paucity of systematic empirical examination validating the complete theoretical chain of perceived value, satisfaction and specific movie consumption behavioral intention. Specifically, the differential indirect effects via satisfaction exerted by distinct types of perceived value (e.g., functional, emotional, social) on varied movie consumption behavioral intention, alongside the precise strength and mechanisms of these pathways, are insufficiently understood. Current models may inadequately capture the complexity of consumer value perceptions and their subsequent impact chains within the movie context (Lee et al., 2016; Tang & Yu, 2021).

Consequently, this study aims to examine the relationships linking perceived value, satisfaction, and movie consumption behavioral intention. The primary objectives include: (1) examining hierarchical effects of value dimensions of consumers’ perceived value within Chinese movie consumption contexts; (2) constructing and empirically testing the direct impact pathways of perceived value on subsequent movie consumption behavioral intention; (3) precisely examining the core mediating role of satisfaction in the aforementioned pathways, thereby revealing how perceived value influences movie consumption behavioral intention via enhanced satisfaction; and (4) providing evidence-informed decision-making support for stakeholders across the film industry value chain (e.g., studios, distributors, exhibitors, streamers). The focus is on value delivery and customer satisfaction management strategies to foster the industry’s sustainable development.

## 2. Literature Review

### 2.1. Perceived Value and Movie Consumption Behavioral Intention

Customer Perceived Value (CPV) is “the consumer’s overall assessment of utility based on perceptions of gains versus sacrifices” (Zeithaml, 1988, p. 14). It manifests through functional (price/quality), social (belonging/identity), and emotional (enjoyment/immersion) dimensions (Sweeney & Soutar, 2001). In movie consumption, which is characterized as an inherently experiential service, these dimensions form a “value blueprint” driving audience choices (Alter, 2013). Critically, the transformation from value perception to behavioral intention involves nonlinear mechanisms mediated by psychological evaluations. While prior studies confirm perceived value’s direct impact on behavioral intention like repurchase (C. Wang et al., 2023), its differential effects across dimensions remain underexplored in movie contexts (Hasson et al., 2008). This perspective aligns with Zeithaml’s (1988) emphasis on the core consumer trade-off. Recognizing movie as an experience-intensive product, scholars have further delineated unique dimensions: notably, the experiential and hedonic value highlighted by Holbrook and Hirschman (1982), alongside the situational value proposed by Sheth et al. (1991), which pertains to viewing environment atmosphere and ritual significance. Empirically, the commercial dominance of movies released during festive seasons like the Lunar New Year demonstrates potent resonance in emotional and social value dimensions, while high-concept spectacle blockbusters leverage their technical prowess primarily within the functional domain (Yang, 2023). Collectively, these value dimensions constitute the fundamental “value blueprint” guiding audience movie selection, serving as the primary catalyst for consumption intention (Alter, 2013).

The transformation from value perception to behavioral intention constitutes a non-linear pathway mediated by multifaceted attitudinal formation and evaluative processes. Consumers develop specific attitudes toward movies (e.g., perceived worthiness) based on value assessments, subsequently shaping behavioral intention—a sequential relationship empirically validated in Gupta et al.’s (2024) investigation of streaming movie consumption. Concurrently, satisfaction emerges as a critical mediating mechanism (Bai et al., 2024); audiences experiencing misalignment between expectations and perceived value exhibit diminished sequel consumption and theater loyalty (H. Kim & Jensen, 2014; E. Kim & Kim, 2017). Recent research highlights three dynamics in value perception: heightened contextual dependence, cultural variability, and digital transformation (Butollo et al., 2022). First, context assumes greater analytical weight, as scholars increasingly frame consumption value not as an absolute attribute but as a provisional construct contingent upon external environmental variables and individual states (Lopez-Orosco et al., 2023). Second, investigation of value’s cultural embeddedness intensifies, recognizing different weighting of functional, emotional, and social values across cultural contexts and regional markets. For example, the distinctive socio–cultural resonance of martial arts cinema among East Asian audiences reflects implicit cultural–symbolic drivers (Jang et al., 2021; A. Kim et al., 2021). Third, digital transformation, particularly video streaming platforms augmented by algorithms, fundamentally reshapes value construction; algorithmic personalization enhances functional utility through time efficiency, but it concurrently risks generating informational filter bubbles that may erode the emotional and experiential satisfaction from diverse genre exploration (Darwich & Bayoumi, 2025). These developments necessitate a paradigm shift beyond static, universalistic value frameworks toward interrogating the dynamic articulation of pluralistic consumption values. Understanding evolving market ecologies and cultural intersections is essential for robust predictive models of movie consumption behavior, offering significant theoretical and practical implications.

### 2.2. Satisfaction and Movie Consumption Behavioral Intention

Satisfaction derives from the disconfirmation between expectations and perceived performance (Oliver, 1980). It directly influences behavioral intention such as repurchase and word-of-mouth (Ghorbanzadeh, 2021). In movie contexts, satisfaction is amplified by emotional engagement and social sharing (Tontini et al., 2022), and directly predicts sequel consumption and premium payment willingness (Báez-Montenegro & Devesa-Fernández, 2017). However, the mediating role of satisfaction between multidimensional perceived value and downstream intention remains undertheorized. Emotional arousal and post-consumption value assessments may amplify this relationship (Yoon, 2018; Zsidó, 2024), yet few studies model these linkages holistically within film contexts. Satisfaction’s influence on behavioral intention within the movie domain is significantly augmented by the medium’s inherently highly experiential nature, substantial emotional engagement requirements, and strong propensity for social sharing, establishing its resultant impact as both particularly salient and complex (Tontini et al., 2022).

Current research shows consensus across diverse intercultural samples and distinct audience segments—such as specialty arthouse viewers and parent–child viewing cohorts—that satisfaction exerts a robust and consistently significant positive influence on behavioral intention in movie consumption (Lee et al., 2016; Teng, 2020). Researchers have enhanced prediction model accuracy by making finer-grained distinctions between satisfaction measurement dimensions, for example, distinguishing between satisfaction from core movie content and peripheral service elements (Xiao et al., 2017). This influence extends not only through direct “satisfaction-consumption” pathways but also frequently through mediating and moderating psychological mechanisms. Increased satisfaction strengthens emotional ties and trust (i.e., customer loyalty), prompting consumers to actively share experiences. This creates a favorable word-of-mouth network and increases the tendency to spread positive comments and reviews on social media platforms, fostering a consumption-promoting network effect (Ghorbanzadeh, 2021; Ghorbanzadeh & Rahehagh, 2020). Empirical evidence further identifies additional variables as critical mediators or amplifiers within this chain—particularly the intensity of emotional arousal during viewing (e.g., tension, enjoyment) and perceived value assessed post-consumption (Zsidó, 2024).

Contemporary research frontiers are progressively refining conceptual frameworks and practical applications through a multidimensional, multi-actor and technologically innovative lens. This enhanced experiential orientation is evident in moving beyond mere cognitive assessment to study enduring structural influences at the emotionally driven level, such as the impact of emotional responses (e.g., empathy, peak experiential states) and sensory memory formation on long-term behavioral patterns (McRae, 2016). Multilevel analyses are now mainstream; research paradigms have expanded from individual consumer-level investigations to comparing East Asian audiences’ emphasis on narrative emotional depth with Western markets’ emphasis on visual spectacle (Tsai & Clobert, 2019), studies of specific community interaction dynamics such as fan subcultures (van Kleef et al., 2019), and analysis of how other stakeholders actively shape behavioral pathways associated with satisfaction (Pratt, 2023). This provides precision decision support for key management practices such as personalized content recommendations, targeted marketing, and reputational risk early warnings (Canavilhas, 2024).

### 2.3. Movie Consumers’ Perceived Value and Satisfaction

Within the domain of consumer behavior research, perceived value and satisfaction stand as pivotal constructs, their intrinsic interconnection constituting the theoretical bedrock for deciphering consumer decision-making and loyalty mechanisms. Rooted in Zeithaml’s (1988) definition, perceived value theory posits a subjective appraisal arising from the consumer’s assessment of perceived gains relative to sacrifices inherent in a transaction. Conversely, the expectancy-disconfirmation model proposed by Rust and Oliver (1994) was considered “as a means to measure customer satisfaction based on the difference between the perceived customer expectation and experience in products or services” (Qazi et al., 2017, p. 451). Foundational theoretical inquiry has long established perceived value as a primary cognitive antecedent driving the formation of consumers’ satisfaction (Parasuraman & Grewal, 2000). Consumers are not passive; they dynamically integrate information related to product attributes, the service environment, and incurred costs to assess subjectively value levels, significantly influencing subsequent satisfaction. This exemplifies the fundamental value-driven satisfaction link.

Extensive empirical inquiry has substantiated and refined the understanding of the relationship mechanisms between these constructs, consolidating a robust scholarly consensus. On the one hand, perceived value has a direct impact on satisfaction: consumers are significantly more satisfied when they perceive extraordinary cost-effectiveness or deep belonging (H. H. Hu et al., 2009). On the other hand, however, the factors that confer the value dimension also exhibit environmental contingencies (Bani-Khaled et al., 2025). Perceived value precedes satisfaction, but this relationship exhibits contextual contingency: high-emotion services prioritize socio-emotional over functional value (Phillips & Baumgartner, 2002). Crucially, prior studies have established that satisfaction mediates the effect of perceived value on repurchase intention and brand loyalty (Tong, 2022). Satisfaction consistently manifests as the essential conduit through which value perceptions enhance higher-order loyalty behaviors. For instance, Mcdougall and Levesque (2000) found that perceived relational value during service encounters indirectly enhances loyalty specifically via strengthened consumer satisfaction.

Within the specific experiential consumption domain of cinema, Pine and Gilmore’s (1999) experience economy framework substantially enriches conceptualization. It delineates multiple facets of cinematic value: functional dimensions (venue accessibility, ticket affordability), affective dimensions (pleasure from emotional resonance), social dimensions (community belonging, cultural identification), and further incorporating esthetic and cognitive interactive elements (Ren et al., 2024). Operationally, perceived movie value materializes through the consumer’s integrated assessment of narrative substance, audiovisual efficacy, star appeal, exhibition quality, and socio-cultural relevance. When cognitively compared against pre-consumption expectations, this assessment fundamentally determines ensuing satisfaction (Jiang et al., 2022). Empirical evidence confirms this hierarchy; emotional value is the strongest satisfaction predictor in experiential domains (Arslanagic-Kalajdzic et al., 2020). Despite demonstrable sectoral variations, one conclusion remains unwaveringly consolidated: satisfaction constitutes the temporal manifestation and psychological carrier of prior value appraisal. It is the most impactful trigger for sustained patronage and positive word-of-mouth (C. L. Wang et al., 2020). Therefore, enhancing consumer satisfaction is not the exhibitor or platform’s ultimate objective; its sustainable achievement fundamentally rests upon the strategic cultivation and delivery of a comprehensive, multidimensional value proposition.

The trajectory of contemporary research on movie consumption reveals two distinct paradigm shifts. First, the purchasing behavior of new consumer generations is often characterized by strong emotional projection, defined as “empathic consumption” where consumers project their own emotions, values, and identities onto a product (Palomba, 2020). Second, the deepening concept of the experience economy has contributed to recognizing new key value dimensions. Investigations increasingly confirm the significance of affective interactive value (manifested through deep audience empathy with characters and narrative engagement) and symbolic social value (related to community identity construction and discursive participation in cultural dialogs) (Ádám & Balázs, 2021).

**Figure 1:**
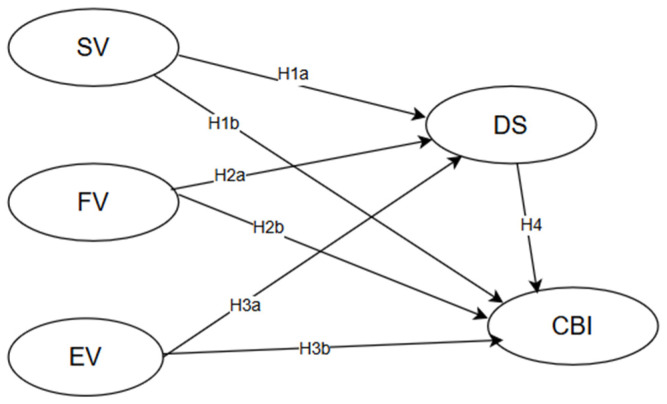
The research hypothesis model.

Informed by the above expansive evidential and conceptual backdrop, the present research developed the following research hypothesis model (Figure 1).

## 3. Methodology

### 3.1. Participants and Procedures

This study employed purposive sampling to strategically select movie consumers capable of providing substantively rich data relevant to research objectives. To gather comprehensive primary evidence through direct engagement with the target demographic, we conducted a 15-day fieldwork phase (25 January to 10 February 2025) involving administered questionnaire distribution across five representative multiplex chains in physical theaters—including Hengdian Cinema, Zhi Chao Theater, and Wanda Cinemas—yielding 359 completed questionnaires. Complementing this approach, the digital reach and connectivity of *Wenjuanxing* Online Platform were leveraged to purposefully target and solicit responses from movie consumers with recent cinema attendance, generating 783 additional submissions. The dual-channel data collection strategy collectively amassed 1142 responses across physical and digital modalities. Following rigorous screening protocols to identify incomplete, inconsistent, or otherwise non-conforming entries, 53 questionnaires were invalidated, resulting in a reliable analytical dataset of 1089 valid responses with a 95.36% valid response rate.

Table 1 presents salient demographic characteristics of the surveyed cohort: First, a predominantly female composition (60.97% of total respondents) aligns with established cultural consumption patterns in China, wherein females demonstrably exhibit stronger inclinations toward cinematic engagement as a leisure pursuit. Second, the age distribution manifests a pronounced concentration (89.90%) below 40 years of age—a patterning highly consistent with the anticipated temporal dynamics of Spring Festival holiday movie attendance, dominated significantly by student populations and younger demographics. Third, educational attainment proves remarkably high, with respondents holding three-year college diplomas or higher constituting 90.18% of the sample, reflecting the empirically observed correlation between elevated cultural consumption and formal education levels among core cinema audiences. Fourth, monthly disposable income predominantly clustered within the 1000–2000 RMB range (61.98%), a distribution intrinsically linked to the substantial representation of student cohorts within the respondent pool, whose typical budgetary constraints correspond to this income bracket.

### 3.2. Data Analysis

The compiled survey data were systematically cataloged into a dedicated database prior to applying statistical procedures. Initial analysis was conducted using SPSS version 26.0, where constructs pertaining to perceived value, satisfaction, and movie consumption behavioral intention were quantitatively assessed; these measures demonstrated adherence to assumptions of normality, thus warranting the implementation of Pearson correlation coefficients to evaluate linear associations. Subsequently, structural equation modeling (SEM) was operationalized via Amos version 26.0 to test the hypothesized relationships. Specifically, the mediation pathway was critically assessed using a bias-corrected self-guided resampling method with 5000 iterations at the 95% confidence level, examining the potential mediating role of satisfaction between perceived value and movie consumption behavioral intention. Indirect effects were inferred to be statistically significant if the resulting confidence intervals did not include zero.

### 3.3. Measures

*Perceived value scale* was conceptualized based on the theoretical foundations established by Zeithaml (1988) and was adapted from the established measurement scales of Sweeney and Soutar (2001). The construct was assessed across three distinct dimensions: social value (3 items), functional value (4 items), and emotional value (4 items), each measured via a 6-point Likert scale ranging from 1 “strongly disagree” to 6 “strongly agree”. These dimensions are particularly relevant to movie consumption, where social value captures shared experiences and identity projection in group settings; functional value addresses tangible aspects like ticketing efficiency and venue amenities intrinsic to cinema services; and emotional value aligns with narrative immersion and affective engagement central to movie appreciation. A sample item is “Watching movie in cinema gave me topics to discuss among friends”. Confirmatory factor analysis (CFA) demonstrated good convergent and discriminant validity for this measurement model, evidenced by key fit indices (χ^2^/df = 3.022; RMSEA = 0.076; CFI = 0.937; TLI = 0.941; NFI = 0.915; SRMR = 0.061), all conforming to established thresholds. Furthermore, the instrument exhibited excellent reliability. The overall scale yielded a Cronbach’s alpha coefficient of 0.913, exceeding the recommended level for internal consistency, which was corroborated by the strong sampling adequacy indicated by a KMO statistic of 0.899 alongside a significant Bartlett’s Test of Sphericity. Subscale analysis confirmed reliability across dimensions, with Cronbach’s alpha coefficients reported as 0.925 (social value), 0.912 (functional value), and 0.913 (emotional value), supported by KMO values of 0.834, 0.802, and 0.807 respectively, collectively affirming the reliability of the scale.

*Satisfaction scale* was operationalized based on conceptual foundations articulated by Oliver (1980, 1997). We defined satisfaction (DS) as the consumer’s psychological assessment of post-viewing affective states arising from movie consumption. This assessment constitutes an evaluative judgment regarding the fulfillment of pre-consumption expectations, wherein satisfaction manifests when perceived performance meets or exceeds anticipations, while dissatisfaction occurs when expectations remain unmet. The construct was measured using a 5-item scale with a 6-point Likert scale ranging from 1 “strongly disagree” to 6 “strongly agree”. Oliver’s expectancy-disconfirmation framework is apposite for movie satisfaction measurement, as audiences form pre-screening expectations regarding narrative coherence, technical execution, and emotional engagement. A sample item is “I had a satisfied experience in the theater setting”. The scale demonstrated excellent internal consistency (Cronbach’s α = 0.940) and strong sampling adequacy (Kaiser-Meyer-Olkin = 0.893), with Bartlett’s Test of Sphericity significant (*p* < 0.001).

*Movie consumption behavioral intention scale* was conceptualized and adapted from Ajzen’s (1991) principles concerning planned behavioral outcomes and Baker and Crompton’s (2000) framework. We defined consumption behavioral intention (CBI) as a post-experiential evaluative construct wherein consumers deliberate to determine future purchase likelihood and associated behaviors. This was measured with a 5-item inventory using 6-point Likert scales (1 = “strongly disagree”, 6 = “strongly agree”). The scale focuses on repurchase likelihood, premium payment willingness, and word-of-mouth, directly mapping to cinema-specific outcomes critical for distributors (e.g., post-screening recommendations, sequel consumption). A sample CBI item is: “I was willing to pay a premium price for movie theater tickets”. The instrument demonstrated robust psychometric properties: excellent internal reliability (Cronbach’s α = 0.919); factorability suitability confirmed by KMO = 0.892 and a significant Bartlett’s Test of Sphericity (*p* < 0.001).

## 4. Results

### 4.1. Common Method Bias Testing

To assess potential common method variance, Harman’s single-factor test was implemented. The unrotated principal component analysis revealed seven components with eigenvalues exceeding the Kaiser criterion threshold of 1.00. Critically, the initial component accounted for merely 26.72% of the total variance, substantially below the conservative 40% benchmark established in methodological literature (Podsakoff et al., 2003). This configuration collectively demonstrates no substantial evidence of systematic method bias contaminating the measurement model.

### 4.2. Descriptive Statistics

Preliminary evaluation of core constructs commenced with descriptive statistics, Kaiser–Meyer–Olkin (KMO) and Bartlett’s sphericity tests, and Cronbach’s alpha reliability analysis. As presented in Table 2, respondents demonstrated elevated attitudinal magnitudes across social value (M = 4.123, SD = 0.971), functional value (M = 4.574, SD = 1.091), emotional value (M = 4.739, SD = 0.931), satisfaction (M = 4.467, SD = 0.928), and behavioral intention (M = 4.378, SD = 1.083), with all means substantially exceeding the theoretical midpoint of 3.00, particularly emotional value manifesting the highest level of recognition. The relatively narrow standard deviation range (0.928 to 1.091) indicates limited dispersion and significant response concentration. Subsequent diagnostics confirmed good measurement properties: the KMO indices for all constructs exceeded 0.80 (range = 0.802–0.893), while Bartlett’s test reached statistical significance (*p* < 0.001), collectively confirming the prerequisites of adequate factorizability and construct validity. Furthermore, consistently excellent Cronbach’s alpha coefficients (minimum α = 0.912 for functional value; maximum α = 0.940 for satisfaction), each substantially exceeding the 0.90 threshold for high internal consistency (Nunnally, 1978), attest to good measurement reliability.

### 4.3. Correlational Analysis

Bivariate correlational analysis (Table 3) revealed statistically significant positive interrelationships across all measured constructs (coefficients ranging r = 0.408–0.691), indicating coordinated covariation between consumer value perceptions (social, functional, emotional), satisfaction, and behavioral intention. The most significant association emerged between functional and social value dimensions (r = 0.691), suggesting consumers’ utilitarian assessments are intrinsically linked with perceived social identity validation. Secondary yet substantial relationships manifested between emotional value and both functional value (r = 0.658) and behavioral intention (r = 0.620), surpassing the satisfaction–behavior linkage (r = 0.571) in magnitude, which is indicative of emotion’s pivotal role in decision architecture. Notably, behavioral intention demonstrated stronger correlations with the three core value dimensions (r = 0.585; 0.589; and 0.620, respectively) than with satisfaction (r = 0.571), implying perceived value constructs offer superior predictive utility relative to satisfaction within the behavioral intention paradigm. While the overall structure is consistent with the previous research finding on the effect of satisfaction (Oliver, 1997), the direct path from emotional value to behavioral intention is particularly strong, which underscores the importance of emotional drivers that need to be strategically acknowledged by movie marketers in order to optimize emotionally driven customer engagement.

### 4.4. Reliability and Validity Testing of Measurement Model

The integrity of measurement instruments fundamentally governs research validity; consequently, SPSS 26.0 and AMOS 26.0 were used for comprehensive reliability and validity assessment. The reliability diagnostics confirmed good internal consistency, with the composite reliability (CR) indices of the structures ranging between 0.901 and 0.936, both of which significantly exceeded the threshold value of 0.70 (Table 4). Content validity was established through systematic adaptation of measurement items from previous literature, ensuring accurate operationalization of movie consumers’ perceived value dimensions, satisfaction, and behavioral intention. Convergent validity evaluation demonstrated excellent measurement properties: all 21 retained indicators exhibited statistically significant factor loadings between 0.75 and 0.95 (well above the 0.70 criterion), while average variance extracted (AVE) estimates for latent constructs ranged from 0.645 to 0.814, surpassing the 0.50 benchmark (Table 4). Discriminant validity was empirically verified as the recommended criteria (Fornell & Larcker, 1981; Teo, 2011), as all square roots of AVEs (diagonal values in Table 3) exceeded corresponding inter-construct correlations in respective rows and columns. Confirmatory factor analysis (CFA) demonstrated a satisfactory fitness for the measurement model, with key indices (χ^2^/df = 2.892; RMSEA = 0.073; CFI = 0.946; TLI = 0.931; and NFI = 0.923; SRMR = 0.057). These integrated assessment evidences attest to the measurement model’s technical adequacy for subsequent structural analyses.

### 4.5. The Fitness Testing of the Final Research Model

This study accounted for age and gender as control variables to address their potential confounding effects on model outcomes. But the supplementary analyses conducted using control variables did not affect the structure of the relationships among the variables. The overall model fitness assessment critically evaluates the correspondence between the hypothesized structural framework and empirical observations, with Table 5 presenting comprehensive diagnostics: absolute fit indices demonstrated χ^2^/df = 2.793 (within acceptable < 3 threshold), RMSEA = 0.073 (satisfactory < 0.08 criterion), and SRMR = 0.056 (<0.06); concurrently, incremental fit measures returned CFI = 0.932, TLI = 0.931 (both exceeding 0.90 convention), alongside NFI = 0.912 and GFI = 0.870—collectively satisfying L.-T. Hu and Bentler’s (1999) joint criteria for adequate specification. This constellation of indices shows theoretically acceptable correspondence between model parameterization and sample covariance structure, thereby justifying proceeding to path coefficient estimation within the structural equation framework.

### 4.6. The Path Analysis of the Structural Equation Model

**Figure 2:**
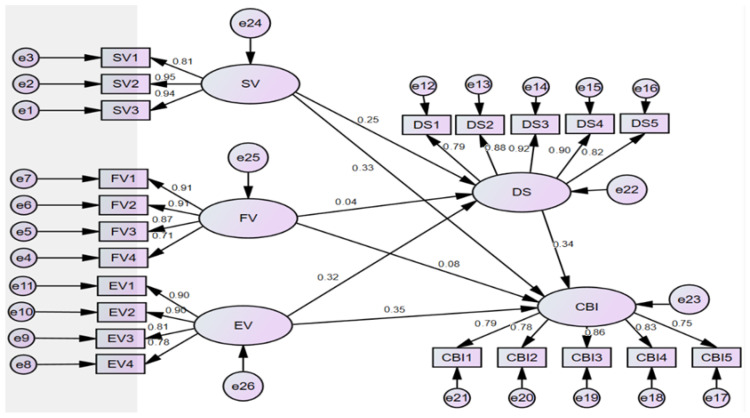
Final structural model.

Path coefficient estimations (see Table 6) revealed standard errors (S.E.) within plausible statistical bounds with no aberrant values. The path analysis of the final structural equation model (Figure 2) confirms statistically significant positive influences of social value (SV: β = 0.246, *p* < 0.001) and emotional value (EV: β = 0.324, *p* < 0.001) on satisfaction (DS), supporting H1a and H3a, which aligns with the multidimensional consumer value theory articulated in Sheth et al.’s (1991) seminal work which posits non-utilitarian dimensions (e.g., relational identity and emotional experiences) as primary satisfaction drivers. Contrastingly, functional value (FV) demonstrated non-significant effects on DS (β = 0.037, *p* = 0.644), suggesting utilitarian attributes may function as baseline requirements rather than distinctive satisfiers in experience-dominant consumption contexts—a phenomenon resonant with Sweeney and Soutar’s (2001) boundary conditions.

Consumer behavioral intention (CBI) emerges through a dual-pathway theoretical framework, wherein satisfaction (DS) operates as a pivotal mediating construct exerting significant influence on CBI (β = 0.337, *p* < 0.001, supporting H4)—thus validating Qazi et al.’s (2017) expectation-confirmation model (ECM) principle positioning satisfaction as the proximal psychological antecedent to behavioral outcomes. Simultaneously, emotional value (EV) and social value (SV) demonstrated substantial direct effects on CBI (EV → CBI: β = 0.351; SV → CBI: β = 0.333; *p*s < 0.001, supporting H1b and H3b), revealing significant direct pathways unmediated by satisfaction that align with Hollebeek and Macky’s (2019) value-to-behavior framework: when consumers derive profound experiential value from emotional connections (EV indicators loading > 0.78) or social identity reinforcement (SV loadings > 0.80), such engagements directly activate behavioral commitment.

In contrast, functional value was a non-significant predictor of CBI (β = 0.077, *p* = 0.249), reconfirming its marginal explanatory role—a finding that is consistent with Parasuraman and Grewal’s (2000) evolutionary theory of technology adoption, which suggests that higher-order value dimensions supersede utilitarian attributes in behavioral decision-making when core functionality is achieved parity across products.

### 4.7. The Mediating Role of Satisfaction

As indicated in Table 7, structural equation modeling analysis examining the interrelationships among perceived value dimensions, satisfaction, and movie consumption behavioral intention (CBI) reveals differential pathways: Social value significantly predicts satisfaction (β = 0.246, *p* < 0.001), which subsequently influences CBI (β = 0.337, *p* < 0.001). Thus, it establishes a significant indirect pathway wherein social value impacts behavioral intention through satisfaction mediation, with the standardized indirect effect quantified at 0.083 (*p* = 0.008; 95% CI [0.030, 0.159]), calculated via bootstrapping. Functionally valued attributes; however, demonstrate statistically insignificant direct effects on satisfaction (β = 0.037, *p* = 0.644) and on CBI (β = 0.077, *p* = 0.249), which did not support H2a and H2b, as well as non-significant mediational impact (β = 0.012, *p* > 0.05). Parallel findings confirm emotional value substantially predicts both satisfaction (β = 0.324, *p* < 0.001) and downstream CBI (β = 0.351, *p* < 0.001), yielding a significant indirect effect (standardized β = 0.109, *p* < 0.001; 95% CI [0.053, 0.189]) that further corroborates satisfaction’s critical mediating role in transforming affective consumer experiences into behavioral outcomes.

## 5. Discussion

This study challenges perceived value dimension universality by demonstrating dominance in socio-emotional factors within movie contexts. Path analyses revealed different causal dynamics: social value (SV) and emotional value (EV) significantly enhance satisfaction (DS), indicating social identity reinforcement and affective resonance are pivotal cinematic satisfaction drivers. Functional value (FV) showed no significant impact. This posits that in high-engagement experiential services, socio-emotional values dominate the value–satisfaction–behavior chain, rendering functional attributes mere hygiene factors (Varshneya & Das, 2017). These findings refine perceived value theory and highlight value hierarchies in experiences.

Substantiating Oliver’s (1997) satisfaction–intention paradigm, DS demonstrated potent direct effects on CBI. Notably, SV and EV evidenced significant direct effects on CBI. The non-significance of the direct effect of functional value on consumption behavioral intention underscores a pivotal finding. Consequently, this study extends the Expectation Confirmation Theory (Oliver, 1980) by demonstrating that confirmation of functional expectations alone is inadequate for satisfaction generation in movie consumption. Instead, satisfaction arises predominantly from the amplification of socio-emotional value dimensions, which reveals an asymmetric disconfirmation mechanism where negative functional performance may cause dissatisfaction, but positive performance yields diminishing returns beyond threshold fulfillment (Behera et al., 2024; Zeelenberg & Pieters, 2004). This redefines the satisfaction calculus in experiential contexts. Irrespective of the mediating mechanisms considered, socio-emotional factors emerge as the predominant determinants in the formation of consumption behavioral intention.

The model empirically establishes emotional value (EV) and social value (SV), though not functional value (FV), as predominant determinants of moviegoer satisfaction and critical predictors of subsequent consumption behavioral intention (CBI), demonstrating satisfaction’s pivotal mediation role while affirming SV and EV as substantive direct predictors of CBI. This study makes a distinctive theoretical contribution by providing a context-specific refinement of perceived value theory within experiential consumption domains, particularly in the cinematic context where socio-emotional engagement is paramount. While perceived value dimensions (functional, emotional, social) are established as antecedents of behavioral intention in consumer behavior literature (Zeithaml, 1988; Sweeney & Soutar, 2001), the findings of this study reveal a hierarchical structure of these dimensions previously underexplored in movie consumption. Specifically, this study demonstrates that emotional value and social value dominate as primary drivers of both satisfaction and direct movie consumption behavioral intention, and that functional value operates as a threshold factor, where its absence may cause dissatisfaction, but its presence does not significantly enhance satisfaction or behavioral intention once baseline expectations are met. This finding refines Parasuraman and Grewal’s (2000) value–satisfaction–behavior framework, thereby advocating strategic prioritization for experience-centric industries.

Path analysis validates satisfaction as the critical conduit through which value dimensions shape movie consumption behavioral intention, with the data revealing statistically significant indirect effects for both emotional value (EV → DS → CBI: βind = 0.109, 95% CI [0.053, 0.189]) and social value (SV → DS → CBI: βind = 0.083, 95% CI [0.030, 0.159]). Dominance within this pathway proves particularly pronounced for EV, manifesting the largest indirect effect magnitude which aligns fundamentally with Kumar’s (2022) experiential consumption framework: Emotional absorption generated through cinematic narratives or esthetic engagement augments satisfaction and consequently activates engagement behaviors like word-of-mouth or repurchase. The non-significant indirect effect of functional value reinforces the proposition that in the current context of movie consumption, functional attributes, such as technological features or scheduling convenience, merely secure baseline acceptance thresholds rather than decisive drive motivation (Gupta et al., 2024).

The dimension-specific mediation efficacy is substantiated through differential effect magnitudes between social value (SV) and emotional value (EV), where EV’s indirect effect marginally eclipsed SV, which validates Jiang et al.’s (2022) dual-value framework in cinematic contexts: Self-oriented emotional fulfillment (e.g., catharsis, narrative absorption) consistently outperforms collective affiliative benefits (e.g., conversational currency, identity signaling) in behavioral conversion efficiency. Compared with the previously demonstrated direct effect on behavioral intention, SV and EV influence movie consumption behavioral intention through dual-path activation (direct push plus satisfaction-mediated indirect path). This indicates that perceived socio-emotional value dimensions operate as primary drivers of behavioral intention in the studied context, highlighting their strategic relevance for film marketing.

While this study yields valuable insights into the pathways linking perceived value, satisfaction, and movie consumption intention, careful consideration must be given to the demographic composition of the sample in interpreting the findings regarding generalizability. The observed dominance of emotional and social value pathways, and the non-significance of functional value, may reflect the priorities and consumption contexts of this specific, younger demographic who constitute a core cinema-going segment. Youthful audiences and students might place paramount importance on social connection (driving SV significance) and emotional escapism (amplifying EV role), potentially attributing lower weight to purely functional features or exhibiting different levels of price sensitivity that diminish FV’s statistical prominence. Older consumers, full-time professionals, or higher-income groups might exhibit differential weights across the value dimensions or express distinct satisfaction triggers and behavioral intention. For example, functional aspects like convenience or premium comfort might hold greater power for time-constrained professionals (Al-Issa et al., 2025). Consequently, the results should be interpreted as strongly indicative of the psychological mechanisms within a crucial segment of the contemporary movie market, namely the youth audience rather than as universally applicable across all cinema consumers. This aligns with industry trends actively targeting younger demographics as primary drivers of theatrical attendance (Palomba, 2020; Tontini et al., 2022). Future research explicitly comparing these pathways across diverse age cohorts and income brackets is essential for validating the broader applicability of the model presented here.

## 6. Conclusions

Based on consumer behavior and perceived value theories, this study dissects the different effects of multidimensional perceived value on movie behavioral intention (CBI), revealing the direct effects of EV and SV on movie consumption behavioral intention and the indirect effects via satisfaction. While functional value (FV) was not confirmed. The findings affirm the experiential nature of cinema consumption, wherein emotional resonance and social connection constitute core competitive dimensions. For practitioners, this suggests: (1) Enhancing immersive narratives and character engagement to amplify emotional permeability; (2) Prioritizing consumer emotional needs in content design; and (3) Acknowledging that social value dimensions (e.g., shared experiences, community identity) significantly drive behavioral outcomes.

Despite the appropriate research methods and procedures used in this study, there are a number of shortcomings in this study. Firstly, the cross-sectional design and the self-reported survey data from a demographically restricted sample (N = 1089), predominantly composed of young students, present a risk of biased statistical conclusions and conclusively restrict generalizability. The limited representation of older age groups, non-student populations, and higher-income brackets challenges the claim of universal applicability of the findings across the entire movie-going public. Secondly, this study exclusively measured behavioral intention toward theatrical movie consumption (e.g., cinema attendance, premium ticket purchases) and did not capture intention for streaming or home-based viewing. Future studies should broaden this scope for cross-channel comparisons. Thirdly, the assessment of common method bias relied primarily on Harman’s single-factor test. Although this approach indicated no severe bias, future studies would benefit from employing complementary statistical techniques (e.g., latent method factor models) and procedural remedies (e.g., temporal separation of measurement) where it is feasible to strengthen methodological rigor. Fourthly, the restriction of this study to investigating participants predominantly from an urban area (sample collection locations) may lead to heterogeneity bias. This suggests caution in generalizing the identified causal structure beyond the urban consumption ecology. Addressing these demographic limitations remains a critical priority for subsequent research.

## Figures and Tables

**Table 1:** Demographic statistics.

**1. Gender**		
Options	Percentage %	Subtotal
Male	39.03%	425
Female	60.97%	664
Valid quantity		1089
**2. Age**		
Options	Percentage %	Subtotal
18–25 years old	68.23%	743
26–40 years old	21.67%	236
41–50 years old	8.26%	90
50 years old and above	1.84%	20
Valid quantity		1089
**3. Level of education**		
Options	Percentage %	Subtotal
Postgraduate	7.46%	81
Bachelor’s	63.82%	695
Three-year college	18.90%	206
High school	8.81%	96
Junior high school and below	1.01%	11
Valid quantity		1089
**4. Occupation**		
Options	Percentage %	subtotal
National public servant	5.69%	62
Company employees	9.92%	108
Public institution employees	12.76%	139
Private entrepreneurs	3.31%	36
Freelancer	7.07%	77
Students in school	61.25%	667
Valid quantity		1089
**5. Monthly disposable income (RMB yuan)**		
Options	Percentage %	Subtotal
8000 and above	7.90%	86
6000–8000	12.95%	141
4000–6000	10.19%	111
2000–4000	6.98%	76
1000–2000	61.98%	675
Valid quantity		1089

**Table 2:** Results of descriptive statistics.

	Mean	SD	KMO and Bartlett’s Test	Cronbach α
SV	4.123	0.971	0.834 ***	0.925
FV	4.574	1.091	0.802 ***	0.912
EV	4.739	0.931	0.807 ***	0.913
DS	4.467	0.928	0.893 ***	0.940
CBI	4.378	1.083	0.892 ***	0.919

Note: SV = Social value; FV = Functional value; EV = Emotional value; DS = Satisfaction; CBI = Consumption behavioral intention. *** *p* < 0.001.

**Table 3:** Results of correlational analysis.

	SV	FV	EV	DS	CBI
SV	(0.902)				
FV	0.691	(0.854)			
EV	0.538	0.658	(0.849)		
DS	0.408	0.414	0.446	(0.863)	
CBI	0.585	0.589	0.620	0.571	(0.803)

Note: Diagonal in parentheses: square root of average variance extracted from observed variables (items); and off-diagonal: correlations between constructs.

**Table 4:** The convergent and discriminant validity of the measurement model.

Latent Variables	Observed Variable	Standardized Factor Loading	CR (>0.7)	AVE (>0.5)	Reference Source
SV	SV1	0.81	0.929	0.814	Zeithaml (1988); Sweeney and Soutar (2001)
	SV2	0.95			
	SV3	0.94			
FV	FV1	0.91	0.914	0.729	
	FV2	0.91			
	FV3	0.87			
	FV4	0.71			
EV	EV1	0.90	0.912	0.721	
	EV2	0.90			
	EV3	0.81			
	EV4	0.78			
DS	DS1	0.79	0.936	0.745	Oliver (1980, 1997)
	DS2	0.88			
	DS3	0.92			
	DS4	0.90			
	DS5	0.82			
CBI	CBI1	0.79	0.901	0.645	Ajzen (1991); Baker and Crompton (2000)
	CBI2	0.78			
	CBI3	0.86			
	CBI4	0.83			
	CBI5	0.75			

**Table 5:** Test results of the overall fitness of the model.

χ^2^/df	SRMR	RMSEA	CFI	TLI	NFI
2.793	0.056	0.073	0.932	0.931	0.912

**Table 6:** The path analysis results.

			Estimate	S.E.	C.R.	*p*
DS	<---	FV	0.037	0.081	0.462	0.644
DS	<---	SV	0.246	0.049	3.721	***
DS	<---	EV	0.324	0.074	4.542	***
CBI	<---	DS	0.337	0.043	6.876	***
CBI	<---	EV	0.351	0.058	5.525	***
CBI	<---	FV	0.077	0.06	1.153	0.249
CBI	<---	SV	0.333	0.038	5.75	***
SV3	<---	SV	0.943			
SV2	<---	SV	0.945	0.031	33.538	***
SV1	<---	SV	0.807	0.033	23.372	***
FV4	<---	FV	0.712			
FV3	<---	FV	0.87	0.071	17.093	***
FV2	<---	FV	0.911	0.067	16.988	***
FV1	<---	FV	0.908	0.071	17.008	***
EV4	<---	EV	0.784			
EV3	<---	EV	0.809	0.058	18.055	***
EV2	<---	EV	0.903	0.059	19.523	***
EV1	<---	EV	0.897	0.059	19.463	***
DS1	<---	DS	0.793			
DS2	<---	DS	0.881	0.056	21.715	***
DS3	<---	DS	0.921	0.055	23.255	***
DS4	<---	DS	0.903	0.057	22.207	***
DS5	<---	DS	0.816	0.062	19.3	***
CBI5	<---	CBI	0.752			
CBI4	<---	CBI	0.827	0.06	19.39	***
CBI3	<---	CBI	0.856	0.058	20.159	***
CBI2	<---	CBI	0.782	0.053	18.165	***
CBI1	<---	CBI	0.791	0.059	18.385	***

*** *p* < 0.001.

**Table 7:** Results of the mediational analysis.

From	β	Mediator	β	To	Standardized Indirect Effect	95% Confidence Interval
SV	0.246	DS	0.337	CBI	0.083 **	[0.030, 0.159]
FV	0.037	DS	0.337	CBI	0.012	[−0.052, 0.082]
EV	0.324	DS	0.337	CBI	0.109 ***	[0.053, 0.189]

** *p* < 0.01; *** *p* < 0.001.

## Data Availability

Data supporting this study can be available on the request from the corresponding author.

## References

[B1-behavsci-16-00556] Ajzen I. (1991). The theory of planned behavior. Organizational Behavior and Human Decision Processes.

[B2-behavsci-16-00556] Al-Issa N., Abiad M., Kallach L., AlAkoum A. (2025). The new luxury equation: Consumer values and market preferences. Cogent Social Sciences.

[B3-behavsci-16-00556] Alter S. (2013). Value blueprint and service design space for facilitating value creation. Nineteenth Americas Conference on Information Systems.

[B4-behavsci-16-00556] Arslanagic-Kalajdzic M., Kadic-Maglajlic S., Miocevic D. (2020). The power of emotional value: Moderating customer orientation effect in professional business services relationships. Industrial Marketing Management.

[B5-behavsci-16-00556] Ádám H., Balázs G. (2021). Movie consumption related trends and countertrends in consumer behavior. SocioEconomic Challenges.

[B6-behavsci-16-00556] Bai S., Sun T., He H. (2024). Exploring the emotional mechanism of consumer satisfaction in new energy vehicles: A dual-path model of intelligent and eco-friendly experiences. Frontiers in Psychology.

[B7-behavsci-16-00556] Baker D., Crompton J. L. (2000). Quality, satisfaction and behavioral intentions. Annal of Tourism Research.

[B8-behavsci-16-00556] Bani-Khaled S., Azevedo G., Oliveira J. (2025). Environmental, social, and governance (ESG) factors and firm value: A systematic literature review of theories and empirical evidence. AMS Review.

[B9-behavsci-16-00556] Báez-Montenegro A., Devesa-Fernández M. (2017). Motivation, satisfaction and loyalty in the case of a film festival: Differences between local and non-local participants. Journal of Cultural Economics.

[B10-behavsci-16-00556] Behera D. K., Rahut D. B., Padmaja M., Dash A. K. (2024). Socioeconomic determinants of happiness: Empirical evidence from developed and developing countries. Journal of Behavioral and Experimental Economics.

[B11-behavsci-16-00556] Butollo F., Gereffi G., Yang C., Krzywdzinski M. (2022). Digital transformation and value chains: Introduction. Global Networks.

[B12-behavsci-16-00556] Canavilhas J., Goyanes M., Cañedo A. (2024). Big data, artificial intelligence and their effects: The birth of an alert society. Media influence on opinion change and democracy.

[B13-behavsci-16-00556] Darwich M., Bayoumi M. (2025). Conclusion and future directions for video streaming enhancements. Enhancing video streaming with AI, cloud, and edge technologies.

[B14-behavsci-16-00556] De Luca T. (2017). Figuring a global humanity: Cinematic universalism and the multinarrative film. Screen.

[B15-behavsci-16-00556] Fornell C., Larcker D. F. (1981). Evaluating structural equation models with unobservable variables and measurement error. Journal of Marketing Research.

[B16-behavsci-16-00556] Ghorbanzadeh D. (2021). From satisfaction to loyalty: The role of emotional structures in the process of transition from satisfaction to loyalty. Asia-Pacific Journal of Business Administration.

[B17-behavsci-16-00556] Ghorbanzadeh D., Rahehagh A. (2020). The role of emotional structures in the relationship between satisfaction and brand loyalty. Cogent Psychology.

[B18-behavsci-16-00556] Gupta S., Deodhar S. J., Tiwari A. A., Gupta M., Mariani M. (2024). How consumers evaluate movies on online platforms? Investigating the role of consumer engagement and external engagement. Journal of Business Research.

[B19-behavsci-16-00556] Hasson U., Landesman O., Oscar B. (2008). Neurocinematics: The neuroscience of film consumption. Projections: The Journal for Movies and Mind.

[B20-behavsci-16-00556] Holbrook M. B., Hirschman E. C. (1982). The experiential aspects of consumption: Consumer fantasies, feelings, and fun. Journal of Consumer Research.

[B21-behavsci-16-00556] Hollebeek L. D., Macky K. (2019). Digital content marketing’s role in fostering consumer engagement, trust, and value: Framework, fundamental propositions, and implications. Journal of Interactive Marketing.

[B22-behavsci-16-00556] Hu H. H., Kandampully J., Juwaheer T. D. (2009). Relationships and impacts of service quality, perceived value, customer satisfaction, and image: An empirical study. The Service Industries Journal.

[B23-behavsci-16-00556] Hu L.-T., Bentler P. M. (1999). Cutoff criteria for fit indexes in covariance structure analysis: Conventional criteria versus new alternatives. Structural Equation Modeling.

[B24-behavsci-16-00556] Hussain R. (2016). The mediating role of customer satisfaction: Evidence from the airline industry. Asia Pacific Journal of Marketing and Logistics.

[B25-behavsci-16-00556] Jang M., Baek H., Kim S. (2021). Movie characteristics as determinants of download-to-own performance in the Korean video-on-demand market. Telecommunications Policy.

[B26-behavsci-16-00556] Jiang X., Deng N., Fan X., Jia H. (2022). Examining the role of perceived value and consumer innovativeness on consumers’ intention to watch intellectual property films. Entertainment Computing.

[B27-behavsci-16-00556] Kang L., Peng F., Sajid Anwar S. (2022). All that glitters is not gold: Do movie quality and contents influence box-office revenues in China?. Journal of Policy Modeling.

[B28-behavsci-16-00556] Kim A., Trimi S., Lee S.-G. (2021). Exploring the key success factors of films: A survival analysis approach. Service Business.

[B29-behavsci-16-00556] Kim E., Kim S. (2017). Online movie success in sequential markets: Determinants of video-on-demand film success in Korea. Telematics and Informatics.

[B30-behavsci-16-00556] Kim H., Jensen M. (2014). Audience heterogeneity and the effectiveness of market signals: How to overcome liabilities of foreignness in film exports?. Academy of Management Journal.

[B31-behavsci-16-00556] Kumar A. (2022). The unmatchable brightness of doing: Experiential consumption facilitates greater satisfaction than spending on material possessions. Current Opinion in Psychology.

[B32-behavsci-16-00556] Lee J., Chen C. C., Song H. J., Lee C. K. (2016). Consumption of movie experience: Cognitive and affective approaches. Journal of Quality Assurance in Hospitality & Tourism.

[B33-behavsci-16-00556] Lopez-Orosco L. A., Solano-Guevara V. A., Turriate-Guzman A. M., Alarcón-Llontop L. R., Shakya S., Papakostas G., Kamel K. A. (2023). Analysis of digital data consumption of video streaming platforms during COVID-19. Mobile computing and sustainable informatics.

[B34-behavsci-16-00556] Mcdougall G. H., Levesque T. J. (2000). Customer satisfaction with services: Putting perceived value into the equation. Journal of Services Marketing.

[B35-behavsci-16-00556] McRae K. (2016). Cognitive emotion regulation: A review of theory and scientific findings. Current Opinion in Behavioral Sciences.

[B36-behavsci-16-00556] Nunnally J. C. (1978). Psychometric theory.

[B37-behavsci-16-00556] Oliver R. L. (1980). A cognitive model of the antecedents and consequences of satisfaction decisions. Journal of Marketing Research.

[B38-behavsci-16-00556] Oliver R. L. (1997). Satisfaction: A behavioral perspective on the consumer.

[B39-behavsci-16-00556] Palomba A. (2020). Consumer personality and lifestyles at the box office and beyond: How demographics, lifestyles and personalities predict movie consumption. Journal of Retailing and Consumer Services.

[B40-behavsci-16-00556] Parasuraman A., Grewal D. (2000). The impact of technology on the quality-value-loyalty chain: A Research agenda. Journal of the Academy of Marketing Science.

[B41-behavsci-16-00556] Phillips D. M., Baumgartner H. (2002). The role of consumption emotions in the satisfaction response. Journal of Consumer Psychology.

[B42-behavsci-16-00556] Pine B. J., Gilmore J. H. (1999). The experience economy: Work is theatre & every business a stage.

[B43-behavsci-16-00556] Podsakoff P. M., MacKenzie S. B., Lee J.-Y., Podsakoff N. P. (2003). Common method biases in behavioral research: A critical review of the literature and recommended remedies. Journal of Applied Psychology.

[B44-behavsci-16-00556] Pratt A. C., Virani T. E. (2023). Film making as a creative ecosystem: The case of soho in London. Global creative ecosystems. Dynamics of virtual work..

[B45-behavsci-16-00556] Qazi A., Tamjidyamcholo A., Raj R. G., Hardaker G., Standing C. (2017). Assessing consumers’ satisfaction and expectations through online opinions: Expectation and disconfirmation approach. Computers in Human Behavior.

[B46-behavsci-16-00556] Ren H., Xu F., Lin G. (2024). Interactive movies: Narrative and immersive experience. Academic Journal of Humanities & Social Sciences.

[B47-behavsci-16-00556] Rust R. T., Oliver R. L. (1994). Service quality: New directions in theory and practice.

[B48-behavsci-16-00556] Sheth J. N., Newman B. I., Gross B. L. (1991). Why we buy what we buy: A theory of consumption values. Journal of Business Research.

[B49-behavsci-16-00556] Sweeney J. C., Soutar G. N. (2001). Consumer perceived value: The development of a multiple item scale. Journal of Retailing.

[B50-behavsci-16-00556] Tang Z., Yu L. (2021). The configurational influence mechanism of film consumption experience on customer satisfaction. Journal of Consumer Behavior.

[B51-behavsci-16-00556] Teng H. Y. (2020). Can film tourism experience enhance tourist behavioural intentions? The role of tourist engagement. Current Issues in Tourism.

[B52-behavsci-16-00556] Teo T. (2011). Factors influencing teachers’ intention to use technology: Model development and test. Computers & Education.

[B53-behavsci-16-00556] Tong M. (2022). Customers’ craft beer repurchase intention: The mediating role of customer satisfaction. International Journal of Food Properties.

[B54-behavsci-16-00556] Tontini G., Montibeler Krause V., da Silva L. F., Vieira F. R., Santos T., Andrade J. (2022). What influences the behavior intention of movie theater customers? Comparing linear and nonlinear points of view. International Journal of Quality and Service Sciences.

[B55-behavsci-16-00556] Tsai J. L., Clobert M., Cohen D., Kitayama S. (2019). Cultural influences on emotion: Established patterns and emerging trends. Handbook of cultural psychology.

[B56-behavsci-16-00556] van Kleef G. A., Gelfand M. J., Jolanda Jetten J. (2019). The dynamic nature of social norms: New perspectives on norm development, impact, violation, and enforcement. Journal of Experimental Social Psychology.

[B57-behavsci-16-00556] Varshneya G., Das G. (2017). Experiential value: Multi-item scale development and validation. Journal of Retailing and Consumer Services.

[B58-behavsci-16-00556] Wang C., Liu T., Zhu Y., Wang H., Wang X., Zhao S. (2023). The influence of consumer perception on purchase intention: Evidence from cross-border E-commerce platforms. Heliyon.

[B59-behavsci-16-00556] Wang C. L., Wang Y., Wei J., Chung H. (2020). Understanding experiential consumption: Theoretical advancement and practical implication. Asia Pacific Journal of Marketing and Logistics.

[B60-behavsci-16-00556] Xiao J., Li X., Chen S., Zhao X., Xu M. (2017). An inside look into the complexity of box-office revenue prediction in China. International Journal of Distributed Sensor Networks.

[B61-behavsci-16-00556] Yang Y. (2023). Festival marketing: Film promotion shaped by traditional Chinese culture—Taking the spring festival comedy film as an example. Advances in Economics, Management and Political Sciences.

[B62-behavsci-16-00556] Yoon S. J. (2018). Social-cultural-psychological perspectives on future word-of-mouth research. Journal of Global Scholars of Marketing Science.

[B63-behavsci-16-00556] Zeelenberg M., Pieters R. (2004). Beyond valence in customer dissatisfaction: A review and new findings on behavioral responses to regret and disappointment in failed services. Journal of Business Research.

[B64-behavsci-16-00556] Zeithaml V. A. (1988). Consumer perceptions of price, quality, and value: A means-end model and synthesis of evidence. Journal of Marketing.

[B65-behavsci-16-00556] Zsidó A. N. (2024). The effect of emotional arousal on visual attentional performance: A systematic review. Psychological Research.

